# Cephalometric study of alterations induced by maxillary slow expansion in adults

**DOI:** 10.1016/S1808-8694(15)30051-3

**Published:** 2015-10-19

**Authors:** Almiro José Machado Júnior, Agrício Nubiato Crespo

**Affiliations:** a Dental surgeon specialized in maxillary functional orthopedics, MSc, Associate Professor at the Systemic Dentistry Society of São Paulo.; b PhD in Medical Sciences, Head of the Ophthalmology-Otolaryngology Department at the Medical School of the Campinas State University - Unicamp.

**Keywords:** Maxilla, Expansion, Cephalometry

## Abstract

Maxilla expansion is a procedure that aims at increasing the maxillary dental arch to correct occlusal disharmony. Largely used in children, its efficacy in adults, when craniofacial growth has attained bone maturity, is controversial. **Aim:** The present study has the objective of evaluating cephalometric modifications resulting from maxilla expansion in adult patients, observing the following linear measurements: facial width, nasal width, nasal height, maxillary width, mandibular width and maxillary molar width. **Material and methods:** The sample was composed of 24 frontal teleradiographs, taken before and immediately after the expansions, from 12 male and female patients aged between 18 years and two months and 37 years and eight months. All patients were submitted to slow expansion of the maxillary bones by means of an appliance used in the technique named “dynamic and functional maxillary rehabilitation”. Wilcoxon paired statistical test was used for related samples with a 5% significance level. **Results:** There was a mean increase of 1.92 mm in nasal width and 2.5 mm in nasal height. As regards the linear measurements maxillary and mandibular width, the mean increase was 2.42 mm and 1.92 mm, respectively. A mean increase of 1.41 mm was found for facial width and 2.0 mm for maxillary molar width, alterations which were statistically significant, the mean time was 5.3 months. **Conclusion:** Based on the results obtained, it may be concluded that the use of maxillary expansion induces increase of the facial measurements studied in adults.

## INTRODUCTION

At the beginning of the digestive process, mastication grinds, humidifies and decreases the size of food particles, producing the food bolus which is then swallowed, ending the oral phase of digestion. This phase, represented by mastication and swallowing, is influenced by dental occlusion and plays a relevant role in human physiological equilibrium.

Dental occlusion is the physical relation between the dental and functional elements of the masticatory system components: superior and inferior dental arches, maxilla, jaw, hyoid bone, tongue, lips, cheek and muscles. It has a direct influence over mastication and swallowing, and indirect impact over respiration and speech^1^, ^2^, ^3^.

Odontology seeks the maintenance of this occlusal equilibrium, prevention and interception of deviations from normality of the stomatognatic system which may occur during an individual’s growth and development. When this equilibrium is not reached, deviations from the occlusal equilibrium set in, resulting in physical alterations named malocclusions.

Malocclusions are caused by hereditary and by extrinsic factors. If little can be done to avoid the hereditary factors, a lot can be done to prevent and treat the extrinsic factors. Among the maneuvers used in the treatment of malocclusions caused by extrinsic factors such as posterior cross bite, maxillary atresia and dental overlapping, maxillary expansion obtained through orthodontic, mechanical orthopedics, functional orthopedics, surgery and a combination of all these resources can be used^3^, ^4^.

Employed during growth, the maxilla expansion has been explained by the separation of the palate center and dental orientation associated with the augmentation of facial structures. There is controversy over the efficacy of maxilla expansion in adults, as soon as craniofacial growth has reached its bone maturity^4^, ^5^, ^6^, ^7^, ^8^.

Thus it is not well understood if there is maxillary expansion in adults and its local, regional effects as well as the way in which the growth of the superior dental arch occurs, if as a result of dental orientation or as an effect of palate center separation, or still bone structures growth adjacent to the maxilla.

The present study aims at evaluating possible cephalometric modifications caused by maxilla expansion in adult patients, observing the following linear measures: facial width, nasal width, nasal height, maxillary width, jaw width and molar-maxillary region width.

## MATERIALS AND METHODS

The sample used in clinical, longitudinal, cohort prospective study was obtained at the dental clinic of the specialization course in orthodontics and functional maxillary orthopedics of the Society of Systemic Odontology of the State of São Paulo.

A total of 12 patients, 11 females and 1 male were selected for sampling the patients that fit the following requirements: over 18 years of age, with maxillary atresia, uni or bilateral cross bite, dental line irregularity, and dental overlapping with maxillary atresia. Patients who had suffered head trauma, with periodontal disease and without the first superior molar were excluded from the sampling.

To expand the maxillary bone we used the technique called “Dynamic and Functional Rehabilitation of the Maxilla” advocated by Vaz de Lima. We used a slow activation device for bilateral expansion of the maxillary bones^9^.

**Figure 1:**
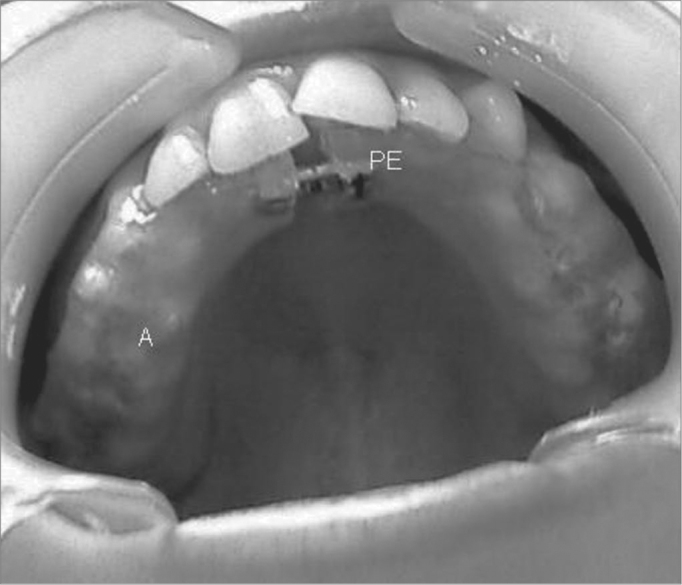
Mouth piece used for maxillary expansion.

The dental piece for maxillary expansion has expanding screws in the palate center projection, wrapped in chemically activated acrylic, covering the whole extension of the hard palate, palate surfaces, and occlusal third of the buccal surfaces of clinical crowns on the posterior teeth (Figure 1).

A= acrylic cover; PE= expander screw.

In placing the dental piece, we verified how the acrylic fits over the hard palate mucosa and posterior teeth surfaces. We asked the patient to remove and replace the dental piece many times, observing any difficulties to remove it, and instructing them to:

1. Use the dental piece constantly, even while sleeping, only removing it for eating and cleaning;
2. Opening the expansion screw: the patient activates the dental piece, rotating ¼ towards the arrow indicating which way it should be done, every other day;
3. Hygiene: the dental piece should be cleaned periodically;
4. Maintenance: every fortnight the patient should return to check dental piece fit, presence of lesions or ulcers, and to make adjustments and polishing when necessary.

How long the patient should use the dental piece for maxilla expansion is yet to be determined. The end of the expansion was established by clinical criteria alone, when we confirmed the correction proposed for each case.

With the goal of evaluating possible cephalometric modifications, all patients were submitted to teleradiographic examinations before the beginning and the end of the treatment described above, with one only equipment, Siemens, Nanomobil model, regulated for exposures of 65 KVp, 10 mA, for 1.5 sec and focal distance of 1.52 meters. We used 18 x 24 cm Kodak x-omat XK1 films.

The radiography, done by a technician, previously calibrated and tested, it was established the position of the head over the cephalometer, in such a way that when the auricular olives are introduced, the patient remains facing the plate and only the nose slightly touches it, being careful so that the medial sagittal plan remains perpendicular to the horizontal plan. We instructed the patient to keep his/hers lips relaxed and in habitual occlusion^10^.

After obtaining all the teleradiography images prior and posterior to treatment we took the measurement of interest for the study. Over each teleradiography image we placed an acetate paper, attached with adhesive tape. Using a negatoscope in a dark room, we delimited the anatomic structures of interest to create the cephalogram: nostril opening, outer skull border, first superior right molar, zygomatic arches, jaw, tuberosity of the maxilla, nasal crest and anterior nasal spine.

**Figure 2:**
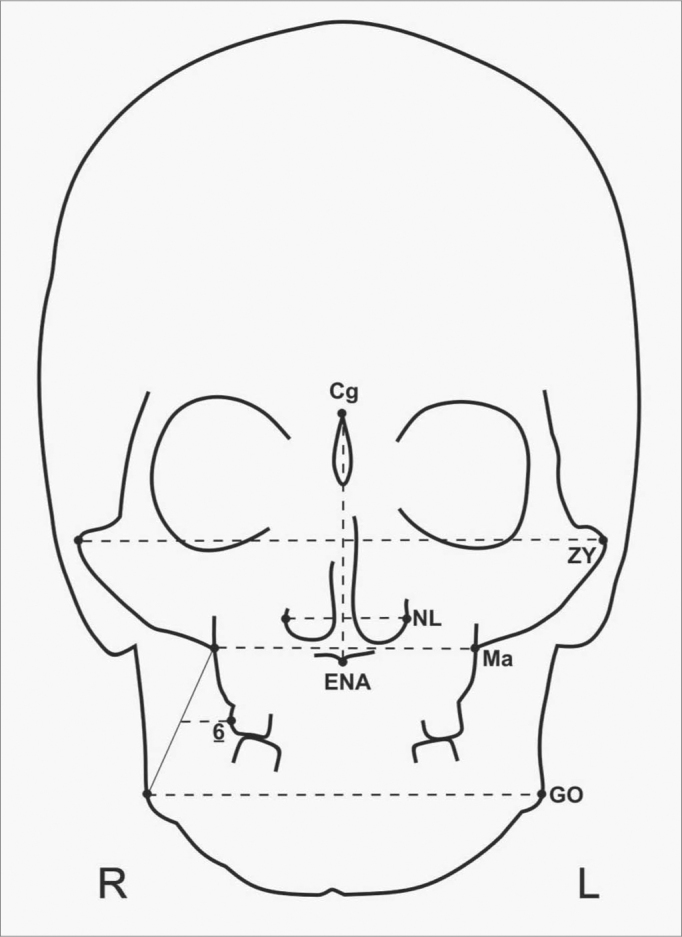
Cephalometric exam by frontal teleradiography.

The following linear measures were taken (Figure 2):

1. Facial width: distance between the bilateral points marked at the outermost tip of the zygomatic arches;
2. Nasal height: distance between the top of the nasal spine and the top of the nasal crest;
3. Nasal width: distance between the outermost external points in the nostril opening;
4. Maxillary width: distance between the two outermost points of the maxillary tuberosities;
5. Jaw width: distance between jaw angles;
6. Molar-maxillary width: distance between the lateralmost point of the first superior right molar crown to the intersection line between the jaw angle, right side, and maxillary tuberosity on, right side.

Cg-ENA=nasal height; ZY=facial width; Ma=maxillary width; NL=nasal width; GO=jaw width; Ma-GO-6=molar-maxillary width.

The observer who did all the measurements of the various distances was previously tested and calibrated, and did not know which patient it belonged to nor if the teleradiography was previous or posterior to the maxillary expansion, in order to avoid biasses. With the goal of minimizing the systematic error and establishing an intraexaminer agreement, each cephalometric measurement was done twice at a 10 day interval, obtaining a level of agreement close to 96%.

In order to compare initial and final measurements, we used the paired non-parametric test of Wilcoxon (for related samples). The level of significance adopted was of 5%. Descriptive statistics of the initial and final measurements (mm), p-value of the paired Wilcoxon are in corresponding tables.

To the patients we presented a signed consent form and the protocol for this research was previously approved by the Ethics Committee in Research of the Unicamp School of Medicine.

## RESULTS

The results are presented in tables showing averages, standard deviations, maximum values, medians and minimal values found for each cephalometric magnitude, time of expansion and age of patients.

Patient age varied between 18 years and two months of age and 37 years and eight months of age, with an average of 24.7, and median of 27 years of age. (Table 1). The time necessary to attain maxillary expansion was of 5.3 months in average, with a standard deviation of 2.57 months, minimum of 3 and maximum of 11 months (Table 2).

**Table 1:** Age of patients, in years, subject to maxillary expansion.

N	AVERAGE	ST. DEV.	MAX	MEDIAN	MIN
12	27.43	5.41	37.08	26.98	18.25

N: number of patients; St. Dev: Standard deviation; MAX: maximum age; MIN: minimum age

**Table 2:** Time for maxillary expansion, in months.

N	AVERAGE	ST. DEV.	MAX	MEDIAN	MIN
12	5.33	2.57	11	5	3

N: number of patients; St. Dev: Standard deviation; MAX: maximum time; MIN: minimum time

Table 3, Table 4, Table 5, Table 6, Table 7, Table 8 show the measurements evaluated before and after the maxillary expansion shown with their averages, Standard deviations, median, minimum and maximum values. All measurements taken show a significant increase after maxillary expansion of around 2.0 mm. The lower increase was observed in facial width, which was 1.41 mm in average (Table 3), and the highest increases observed were on nasal height (2.50 mm in average) and maxillary width (2.42 mm in average) (Tables 5 and 6).

**Table 3:** Cephalometric values of the face width obtained before and after maxillary expansion, in mm.

FACIAL WIDTH						
	N	AVERAGE	ST. DEV.	MAX	MEDIAN	MIN
INITIAL	12	128.67	7.41	144	129	115
FINAL	12	130.08	7.50	146	130	119

N: number of patients; St. Dev: Standard deviation; MAX: maximum value; MIN: minimum value

Wilcoxon test:p-value=0.0078

**Table 4:** Cephalometric values of nasal width obtained before and after maxillary expansion, in mm.

NASAL WIDTH						
	N	AVERAGE	ST. DEV.	MAX	MEDIAN	MIN
INITIAL	12	30.00	2.52	34	29.0	26
FINAL	12	31.92	2.50	35	31.5	26

N: number of patients; St. Dev: Standard deviation; MAX: maximum value; MIN: minimum value

Wilcoxon test :p-value=0.0010

**Table 5:** Cephalometric values of nasal height obtained before and after maxillary expansion, in mm.

NASAL HEIGHT						
	N	AVERAGE	ST. DEV.	MAX	MEDIAN	MIN
INITIAL	12	59.83	4.53	67	59.0	54
FINAL	12	62.33	4.66	69	62.5	56

N: number of patients; St. Dev: Standard deviation; MAX: maximum value; MIN: minimum value

.Wilcoxon test :p-value =0.0020

**Table 6:** Cephalometric values of maxillary width obtained before and after maxillary expansion, in mm.

MAXILLARY WIDTH						
	N	AVERAGE	ST. DEV.	MAX	MEDIAN	MIN
INITIAL	12	65.25	3.70	70	66.5	57
FINAL	12	67.67	4.16	74	67.5	59

N: number of patients; St. Dev: Standard deviation; MAX: maximum value; MIN: minimum value

.Wilcoxon test :p-value =0.0034

**Table 7:** Cephalometric values of jaw width obtained before and after maxillary expansion, in mm.

JAW WIDTH						
	N	AVERAGE	ST. DEV.	MAX	MEDIAN	MIN
INITIAL	12	84.08	4.48	92	83.5	77
FINAL	12	86.00	4.68	95	86.5	79

N: number of patients; St. Dev: Standard deviation; MAX: maximum value; MIN: minimum value

.Wilcoxon test :p-value =0.0010

**Table 8:** Cephalometric values of molar-maxillary width obtained before and after maxillary expansion, in mm.

MOLAR-MAXILLARY WIDTH						
	N	AVERAGE	ST. DEV.	MAX	MEDIAN	MIN
INITIAL	12	5.08	1.62	8	5.0	3
FINAL	12	7.08	1.78	9	7.5	5

N: number of patients; St. Dev: Standard deviation; MAX: maximum value; MIN: minimum value

Wilcoxon test :p-value =0.005

## DISCUSSION

Maxillary expansion has been used to treat malocclusion growth in patients of various ages^6^, ^11^, ^12^, ^13^, ^14^, ^15^, ^16^, ^17^, ^18^.

Studies state that the post-natal growth reaches its peak in middle adolescence and is dramatically reduced at the end of this period. The average age for growth decrease is around 14 years of age for women, and 16 years of age for men^19^, ^20^, ^21^, ^22^, ^23^.

Thus, using the biological concept defined for adult patients, we included in this study patients above 18 years of age, subject to slow maxillary expansion.

In researching the pertinent literature, no mention over the cephalometric observations caused by maxillary expansion in adult patients was found, such fact led to this study.

Cephalometry by frontal teleradiography, because of its ease of use and availability, has been used in anatomic studies and in diagnosing malocclusion^10^.

It has also been used in the observation of alterations caused by induced maxillary expansion^8^, ^24^, ^25^, ^26^, ^27^, ^28^, ^29^, ^30^, ^31^, ^32^, ^33^.

There is controversy over the types of mouth pieces used for rapid maxillary expansion. Various types of diagrams are presented in the literature, but all are constituted basically of an expander screw placed transversally to the palate, differing only on the anchor type used^26^, ^33^, ^34^, ^35^, ^36^.

Some researchers support the use of an acrylic resin, covering the hard palate, offering the mouth piece a tooth-mucosa-supported anchor, to provide better support, favoring the expansion and contention, mainly for the bone^37^, ^38^, ^39^.

For other authors, the mucosal support makes it harder to clean the resin-mucosa interface, and it also causes ulcers and eritematous lesions in the palate mucosa due to the contact and the pressure from the support, and these authors end up choosing a tooth-supported mouth piece^34^.

To attain slow maxillary expansion, we used a tooth-mucosa-supported mouth piece, expecting that the expansion forces would act not only over the posterior teeth, but also and mainly, over the maxillary bone structures^37^, ^38^, ^39^, ^40^.

In this study we used the mouth piece for the slow maxillary expansion, with activation every other day, believing that better results are obtained when the activation, and consequently, the liberation of the expansionary forces are applied intermittently over the maxillary bones^4^, ^39^, ^40^, ^41^.

Even though we did not aim at observing the maintenance of linear measures in the long term after maxillary expansion, we believe the recurrence index, if any, would be lower in the cases where rapid maxillary expansion is used. Still, further studies are necessary to test this hypothesis.

Applied in children, maxillary expansion is controversial in adults, as the craniofacial growth has reached its bony maturity^4^, ^6^, ^7^, ^8^, ^41^.

As to the mechanism of maxillary expansion in adult patients, our results do not support the hypothesis that maxillary expansion occurred due to dental tilting, as suggested in the literature^28^, ^42^, ^43^, ^44^.

If this hypothesis were right there should have been a decrease in molar-maxillary distance, which was not observed in our cases where, on the contrary, there was an average increase of 2.0 mm (Table 8).

Some who use maxillary expansion attribute it to the separation of the palate center^40^, ^42^, ^44^, ^45^, ^46^.

With the methodology used in this study, we did not observe the behavior of palate suture, thus our findings do not allow us to define if the expansion occurred due to palate center separation.

On the other hand, the found average growth of 1.41 mm on facial width (table 3) and 1.92 mm on jaw width (table 7), measurements limited to the maxilla, have led us to suppose that if there is separation of palate center, it is not the only factor leading to maxillary expansion.

Despite the reduced sample size, the results of this study show that the use of maxillary expanders in adults allows for statistically significant expansion, observed by the average increase in the linear measures of facial width (1.41 mm), nasal width (1.92 mm), nasal height (2.5 mm), maxillary width (2.42 mm), jaw width (1.92 mm) and molar-maxillary width (2.0 mm) (Table 3, Table 4, Table 5, Table 6, Table 7, Table 8), in an average time frame of 5 months (table 2).

For the stomatognatic system the expansion movements might be considered fewer than two aspects: induced by mouth pieces which use mechanical forces and mouth pieces which use functional forces. The mechanical movements are a result of forces applied over the teeth and transmitted to the bones, aiming at changing growth direction. The functional movements use natural forces originated from the muscular movement, acting over the velocity and direction of growth and bone remodeling^2^, ^4^.

The maxillary expansion mouth pieces used in this study produce mechanical forces, but their effects produced by expansion such as increase in the mouth cavity, observed on the maxillary width and jaw width, allowing for extra space for the tongue functionality (swallowing, mastication, speech) and increase in the nasal measurements, nasal width and height, allowing for better anatomic functional improvements or nasal respiration, associated with the occlusive alteration, caused by the acrylic cover over the posterior teeth, showed factors which might fit the characteristics of functional movements, meaning, that maxillary expanders produce mechanical movements, allowing subsequent or concomitant functional movements, caused by the increase in mouth cavity size. Still, other studies are necessary to evaluate probable alterations in functionality (swallowing, mastication, and breathing) of the stomatognatic system after maxillary expansion.

We may state that maxillary expansion represent a therapeutic approach inserted with coherence in corrective practices of occlusal deviations, regardless of occlusion stage, as long as the maxillary atresia is part of the morphological deviation. The increase in transversal dimension between the maxillary bones, with an increase in bone mass, is a fact, with great changes in the morphology of the superior dental arch, bringing indisputable advantages in mechanotherapy for maxillary deficiencies.

The results found show the real possibility of maxillary expansion in adult patients, not being restricted to dental orientation or separation of palate center, but also playing a relevant role in the enlargement of facial structureso by maxillary induced expansion.

## CONCLUSIONS

The results obtained in this study allow us to conclude:

• There is maxillary expansion in adults.
• Maxillary expansion leads to an increase in facial, nasal, maxillary, jaw and molar-maxillary widths, and nasal height.
